# Targeted locus amplification to detect molecular markers in mantle cell and follicular lymphoma

**DOI:** 10.1002/hon.2864

**Published:** 2021-03-31

**Authors:** Elisa Genuardi, Petra Klous, Barbara Mantoan, Daniela Drandi, Martina Ferrante, Federica Cavallo, Beatrice Alessandria, Irene Dogliotti, Daniele Grimaldi, Simone Ragaini, Michele Clerico, Mariella Lo Schirico, Elona Saraci, Mehmet Yilmaz, Gian Maria Zaccaria, Sergio Cortelazzo, Umberto Vitolo, Stefano Luminari, Massimo Federico, Mario Boccadoro, Max van Min, Erik Splinter, Marco Ladetto, Simone Ferrero

**Affiliations:** ^1^ Department of Molecular Biotechnology and Health Sciences Division of Hematology University of Torino Torino Italy; ^2^ Cergentis B.V. Utrecht The Netherlands; ^3^ Division of Hematology 1 AOU “Città della Salute e della Scienza di Torino” Torino Italy; ^4^ European Myeloma Network Onlus Torino Italy; ^5^ Oncology Unit Humanitas/Gavazzeni Clinic Bergamo Italy; ^6^ Department of Oncology Division of Hematology AOU Città della Salute e della Scienza di Torino Torino Italy; ^7^ Hematology Unit Azienda USL IRCCS di Reggio Emilia, Reggio Emilia Modena Italy; ^8^ Medical Oncology, CHIMOMO department University of Modena and Reggio Emilia Modena Italy; ^9^ Division of Hematology Azienda Ospedaliera SS Antonio e Biagio e Cesare Arrigo Alessandria Italy

**Keywords:** follicular lymphoma, mantle cell lymphoma, marker screening, minimal residual disease, NGS

## Abstract

Minimal residual disease (MRD) monitoring by PCR methods is a strong and standardized predictor of clinical outcome in mantle cell lymphoma (MCL) and follicular lymphoma (FL). However, about 20% of MCL and 40% of FL patients lack a reliable molecular marker, being thus not eligible for MRD studies. Recently, targeted locus amplification (TLA), a next‐generation sequencing (NGS) method based on the physical proximity of DNA sequences for target selection, identified novel gene rearrangements in leukemia. The aim of this study was to test TLA in MCL and FL diagnostic samples lacking a classical, PCR‐detectable, *t*(11; 14) MTC (BCL1/IGH), or *t*(14; 18) major breakpoint region and minor cluster region (BCL2/IGH) rearrangements. Overall, TLA was performed on 20 MCL bone marrow (BM) or peripheral blood (PB) primary samples and on 20 FL BM, identifying a novel BCL1 or BCL2/IGH breakpoint in 16 MCL and 8 FL patients (80% and 40%, respectively). These new breakpoints (named BCL1‐TLA and BCL2‐TLA) were validated by ASO primers design and compared as MRD markers to classical IGH rearrangements in eight MCL: overall, MRD results by BCL1‐TLA were superimposable (R Pearson = 0.76) to the standardized IGH‐based approach. Moreover, MRD by BCL2‐TLA reached good sensitivity levels also in FL and was predictive of a primary refractory case. In conclusion, this study offers the proof of principle that TLA is a promising and reliable NGS‐based technology for the identification of novel molecular markers, suitable for further MRD analysis in previously not traceable MCL and FL patients.

## 1 INTRODUCTION

Mantle cell (MCL) and follicular lymphoma (FL) are B‐cell non‐Hodgkin lymphomas (NHL) characterized by the *t*(11; 14) and *t*(14; 18), translocation causing BCL1 or BCL2/IGH rearrangements.^1^ Despite considerable therapeutic progress, eventually both cancers tend to relapse, even after long periods of clinical remission.^2^, ^3^ To predict disease relapse, it is necessary to early assess the presence of residual clonal cells after a successful treatment. Therefore, minimal residual disease (MRD) analysis, based on the highly sensitive PCR, for BCL1 and BCL2/IGH^4^, ^5^, ^6^ and IGH clonal rearrangements detection^7^, ^8^ has become a widely used and standardized approach to monitor the disease persistence.^9^ Overall, current standard PCR techniques allow the identification of a molecular marker in 75%–80% of MCL patients, namely 65%–70% carrying an IGH clonal rearrangement and up to 30%–35% an amplifiable BCL1/IGH rearrangement (with a breakpoint located in the major translocation cluster [MTC] region ).^10^, ^11^ In FL patients, a molecular marker, either BCL2/IGH major breakpoint region (MBR) or minor cluster region (MCR), is available in 55%–60% of affected cases.^12^, ^13^, ^14^ Thus, about 20% of MCL and 40% of FL cases lacking a molecular marker are currently still not eligible for MRD assessment with the classical PCR techniques (i.e., “no marker patients”).

Actually, the introduction of next‐generation sequencing (NGS) opened new perspectives in this context.^15^, ^16^ The recently developed targeted locus amplification (TLA) technology is able to sequence structural variants, usually not detected by conventional PCR approaches.^17^ TLA, by selective amplification and sequencing of entire genes on the basis of the crosslinking of physically proximal DNA loci, was able to identify novel candidate oncogenes and not yet described gene fusion partners in B‐ and T‐cell acute lymphoblastic leukemia (ALL), increasing the knowledge of genomic diversity and driving oncogenic lesions.^18^

The aim of this study was to apply TLA on samples collected from MCL and FL patients enrolled in prospective clinical trials of the Fondazione Italiana Linfomi (FIL) and lacking a PCR‐detectable BCL1 (MTC) or BCL2 (MBR and MCR) rearrangement, in order to assess the feasibility of TLA as marker screening approach. In both sample populations, new BCL1 and BCL2/IGH breakpoints (namely “BCL1‐TLA and BCL2‐TLA”) were addressed and their performance as MRD markers was evaluated. Additionally, MRD levels were compared by ASO‐qPCR in those MCL cases with an already available IGH clonal rearrangement. Finally, MRD trends were monitored in MCL and FL “no marker” patients, when follow‐up samples were available.

## 2 METHODS

### 2.1 Patients and samples

Patients affected from MCL and FL enrolled in prospective clinical trials of the Fondazione Italiana Linfomi (FIL) (NCT02354313, EudraCT 2012‐003170‐60, 2012‐001676‐11, and 2012‐000251‐14), were selected for this study. Bone marrow (BM) and peripheral blood (PB) samples were collected at diagnosis (baseline) and during different clinical follow‐up (FU) time‐points, as scheduled by the trials. In MCL samples, multicolor flow cytometry (MFC) was performed both on BM and PB, in order to quantify tumor infiltration rate, using the recommended Euro‐Flow Consortium.^19^

The clinical trials, as well as the MRD study, were approved by the ethical committees of all the enrolling centers. All patients provided written informed consent for the use of their biological samples for research purposes, in accordance with Institutional Review Boards requirements and the Helsinki's declaration.

### 2.2 IGH and BCL1 and BCL2/IGH marker screening and MRD monitoring by PCR

Mononuclear cells (MNCs) were isolated from PB and total white blood cells (WBCs) from BM using Ficoll Hystopaque (Sigma‐Aldrich/Merck) stratification and red blood cells lysis procedure (NH_4_Cl solution, pH 7), respectively, as previously described.^20^ Housekeeping gene control amplification (i.e., TP53 exon 8) was performed to assess gDNA amplification quality.^21^

MCL diagnostic samples were investigated for IGH gene rearrangements and BCL1/IGH MTC by qualitative PCR and Sanger sequencing.^5^, ^6^, ^7^ FASTA files alignment was performed by IMGT/V‐QUEST (http://imgt.org)^22^ and BlastN tool (NCBI, https://blast.ncbi.nlm.nih.gov/Blast.cgi), in order to set quantitative MRD Polymerase Chain Reaction (PCR) (ASO qPCR),^8^ then analyzed according to the EuroMRD guidelines^9^

### 2.3 Targeted locus amplification

TLA was carried out starting from 3–5 × 10^6^ cells or 5 μg of gDNA.^23^ Circular TLA template (of on average 2 kb in size) was created and amplified with IGH enhancer primers (AGCAATTAAGACCAGTTCCC and CTCCACAACCTCTGAATGG; Cergentis), then indexed by Nextera XT transposon‐based technology and sequenced on MiSeq platform (Illumina).

Sequence reads were mapped against the human genome version hg19 using BWA‐SW, which is a Smith‐Waterman alignment tool. This allows partial mapping, which is optimally suited for identifying break‐spanning reads. Then, the Integrated Genomic Viewer (IGV; http://software.broadinstitute.org/software/igv/) tool was used to identify the mated nucleotide sequences. Moreover, to better define the genomic positions, IGV data were further aligned to the latest published reference genome (GRCh38/hg38) using available free sources tool, as Basic Local Alignment Search Tools (Nucleotide BLAST; https://blast.ncbi.nlm.nih.gov/Blast.cgi) and BLAST‐Like Alignment Tool (BLAT; http://genome.ucsc.edu/cgi‐bin/hgBlat).

To define the exact genomic position of the newly identified BCL1 and BCL2‐TLA breakpoints, an internal lab series of classical MTC, MBR, and MCR breakpoints was used as control and realigned with Clustal Omega—Multiple Sequence Alignment (www.ebi.ac.uk/Tools/msa/clustalo/). Circos software package was used to graphically describe the physical relationships among the translocated loci.^24^

### 2.4 TLA molecular markers sequences validation and MRD monitoring

ASO primers were designed on the BCL1 and BCL2‐TLA juxtaposed chromosomic breakpoints.^25^ MRD quantification was performed on FU samples, according to the Euro MRD criteria.^9^

The correlation between the classical IGH and the BCL1‐TLA molecular markers in MCL samples was performed using bivariate Pearson's regression analysis: quantitative discordances between the two molecular markers were defined as “minor” if one target was positive and the other positive not quantifiable (PNQ)^9^ and “major” if a positive versus negative result occurred.

## 3 RESULTS

### 3.1 Patients and samples features

Overall, TLA was tested in 8 BM and 12 PB diagnostic samples recovered from 20 MCL patients. Median tumor infiltration values were 42% (range 11%–78%) and 44% (range 0.03%–95%) by flow, for BM and PB, respectively.

Among these, 15 out of 20 (75%) patients carried a clonal IGH rearrangement detected by Sanger sequencing, too. Thus, 5 out of 20 patients (25%) were considered “no marker MCL” by conventional molecular biology methods (Table 1).

**TABLE 1:** Patients features

Patients feature**s**	MCL	FL
Analyzed tissue		
BM	8/20 (40%)	23/23 (100%)
PB	12/20 (60%)
Median tumor infiltration by flow (range)		
BM	42% (11%–78%)	nd
PB	44% (0.03%–95%)	nd
Clonal rearrangements detected by PCR	15/20 (75%)	3/23 (13%)
“no marker patients” by PCR	5/20 (25%)	20/23 (100%)

Abbreviations: BM, bone marrow; FL, follicular lymphoma; MCL, mantle cell lymphoma; PB, peripheral blood.

Moreover, 23 BM samples of FL patients were tested by TLA, too. In this group, 20 out of 23 (87%) patients resulted in BCL‐2/IGH (both MBR and MCR) negative by PCR, thus considered “no marker FL” (Table 1). Otherwise, 3 out of 23 (13%) patients had a well‐characterized MBR translocation by classic quantitative PCR approach, with very low tumor burden levels (1E−05 and 1E−06 or positive but not quantifiable, according to EURO‐MRD qPCR guidelines).

Additionally, DOHH2 cellular line, harboring MBR breakpoint, and four healthy donors were tested as positive and negative controls, respectively.

### 3.2 TLA feasibility

**FIGURE 1:**
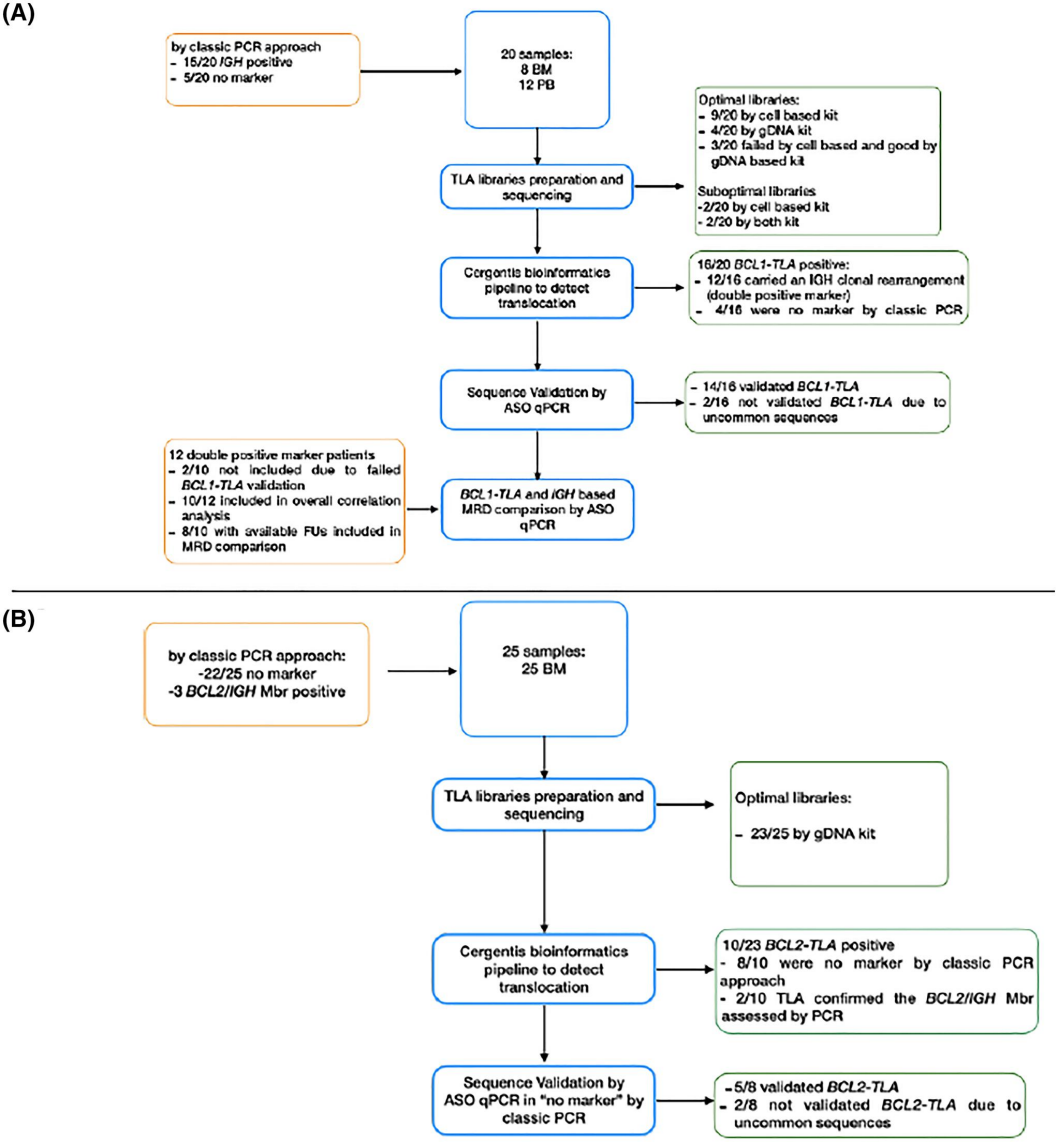
Study workflow and results overview

Overall, TLA enabled the identification of a new *t*(11; 14) breakpoint (“BCL1‐TLA”*)*, in 16 out of 20 patients (80%). Of note, 12 out of these 16 patients (75%) also carried a clonal IGH rearrangement and were thus defined “double positive marker.” Interestingly, new *t*(11; 14) breakpoints were also identified in four out of five (80%) “no marker” MCL patients (Figure 1). Notably, the four patients without a detectable BCL1‐TLA (i.e. TLA 1, TLA14, TLA15, and TLA17) were uniformly characterized by low tumor infiltration (less than 5% by MFC). Therefore, 23 patients were available for the bioinformatics pipeline (20 “no marker” and 3 low tumor burden cases). Notably, among the “no marker” patients, a new *t*(14; 18) molecular marker (BCL2‐TLA) was found in 8 out of 20 (40%) FL samples.

Additionally, the MBR‐positive control sample (DOHH2 cell line) was tested and correctly sequenced, as well. Among the three FL cases with a very low tumor burden (TLA37, TLA39, and TLA42), TLA identified the BCL2‐MBR translocation only in the patient (TLA42) with a quantifiable disease (1E‐05), and no rearrangements were found in those two cases defined as positive but not quantifiable (1E−06 or PNQ).^9^

Finally, as expected, in all the four healthy donors, no rearrangement was found.

### 3.3 Chromosomal breakpoints detection

**FIGURE 2:**
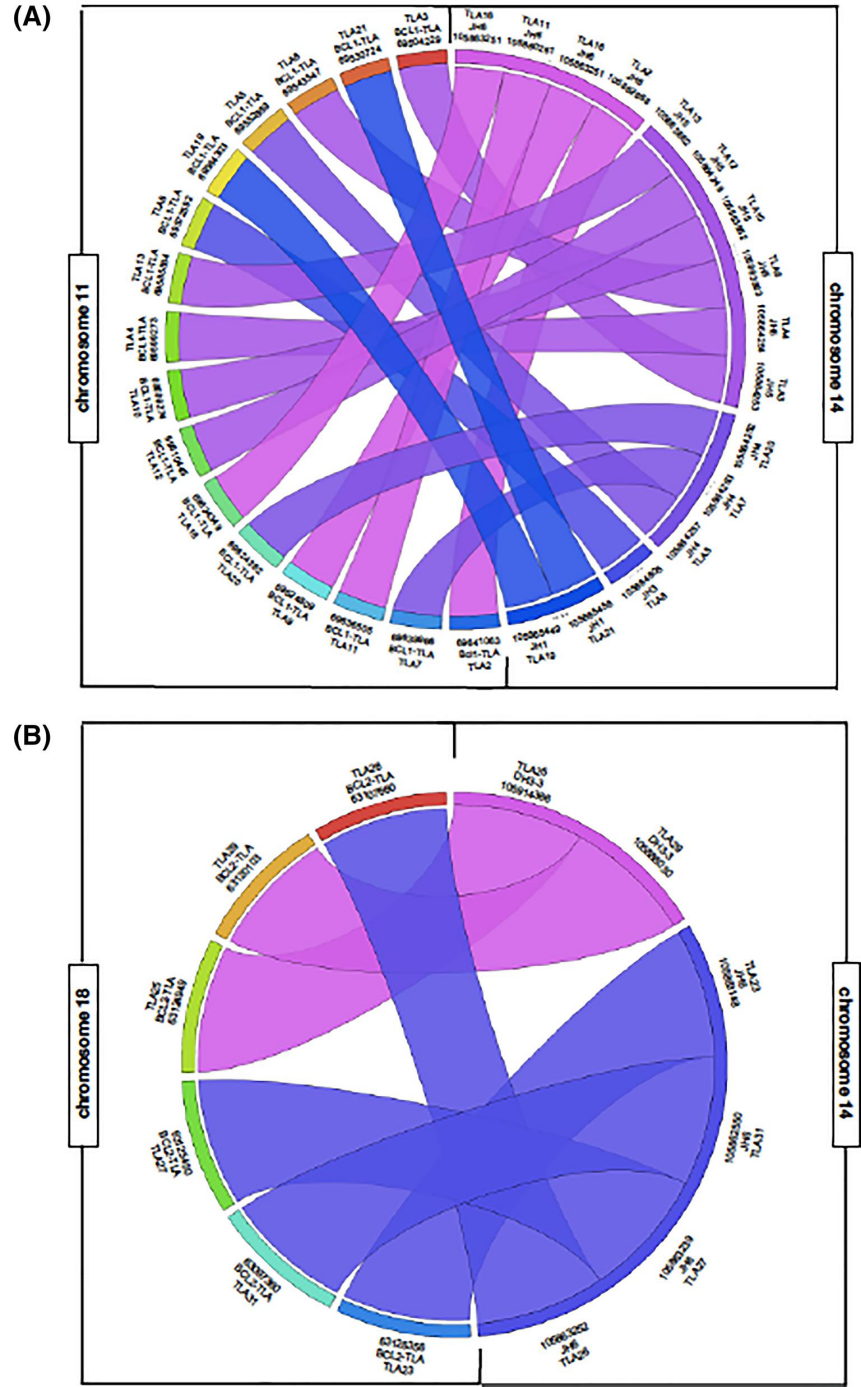
Physical relationship among the translocated loci represented by Circos

**FIGURE 3:**
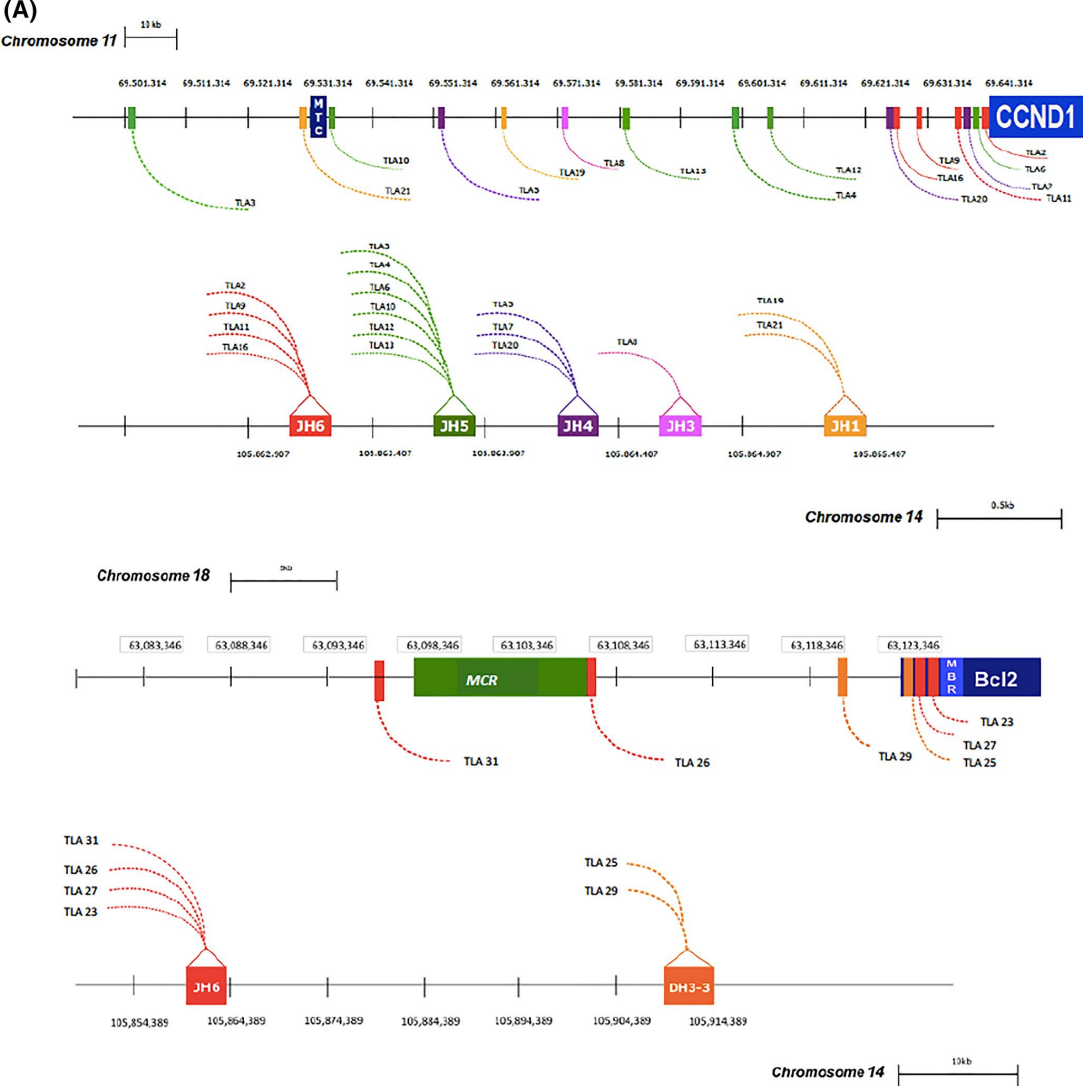
Schematic representation of mated chromosomic regions compared to *MTC*, *MBR*, and *MCR* breakpoint genomic localization. MTC, major translocation cluster; CCND1, cyclin D1 gene; MBR, major breakpoint region; MCR, minor cluster region

To define the exact chromosomal breakpoints regions, binary alignment map (BAM) files were aligned to the reference genome. In MCL samples, clonal reads were identified on chromosome 11 at the 3′ end of the BCL1 locus (ranging the genome nucleotide positions 69503679–69641063), translocated with different IGHJ regions, and mapped on chromosome 14 (annotation:105859639–105865458) (Figure 2a; Table 2). Interestingly, all the newly identified breakpoints were scattered along a 137 kb region on chromosome 11 (with a putative minor cluster right at 3′ of the CCND1 gene, in for four cases TLA2, TLA6, TLA7, and TLA11) and did not involve the classical MTC locus (Figure 3a). Moreover, no preferential usage of a specific IGHJ segment on the chromosome 14 was observed.

**TABLE 2:** BCL1‐TLA and BCL2‐TLA positive samples description

Sample	Tissue analyzed	Tumor infiltration (%)	Sanger	TLA	chr11 start (genome position)	chr11 end (genome position)	bp at 3'side: CCND1	chr14 start (genome position)	chr14 end (genome position)	JH
TLA2	BM	60	IGH	BCL1‐TLA	69640529	69641063	251	105863253	105862668	JH6
TLA3	BM	65	IGH	BCL1‐TLA	69503685	69504229	137407	105863690	105864253	JH5
TLA4	PB	70	IGH	BCL1‐TLA	69598743	69599273	42039	105863690	105864254	JH5
TLA5	PB	71	IGH	BCL1‐TLA	69552333	69552869	88500	105863692	105864257	JH4
TLA6	PB	26	IGH	BCL1‐TLA	69643001	69543547	97767	105863300	105863863	JH5
TLA7	BM	52	IGH	BCL1‐TLA	69639411	69639966	1348	105863691	105864263	JH4
TLA8	BM	17	IGH	BCL1‐TLA	69572005	69572592	68720	105864605	105865113	JH3
TLA9	PB	78	IGH	BCL1‐TLA	69624506	69624809	16269	105860944	105861521	JH6
TLA10	PB	95	IGH	BCL1‐TLA	69598743	69599274	98266	105863792	105863852	JH5
TLA11	PB	56.5	IGH	BCL1‐TLA	69636004	69636505	4809	105859639	105860261	JH6
TLA12	BM	78	IGH	BCL1‐TLA	69609945	69610445	30869	105863697	105864249	JH5
TLA13	BM	59	no marker	BCL1‐TLA	69585008	69585564	55750	105863301	105863862	JH5
TLA16	PB	73	no marker	BCL1‐TLA	69624288	69624349	16965	105863190	105863251	JH6
TLA19	PB	6,5	IGH	BCL1‐TLA	69564262	69564303	77011	105865357	105865449	JH1
TLA20	BM	11	no marker	BCL1‐TLA	69624289	69624362	16965	105864193	105864252	JH4
TLA21	BM	35	no marker	BCL1‐TLA	69530667	69530724	110590	105865413	105865458	JH1

Sample	Tissue analyzed	Sanger	TLA	chr18 start (genome position**)**	chr18 end (genome position)	bp at 3'side: Bcl2	chr14 start (genome position)	chr14 end (genome position)	JH
TLA23	BM	No marker	BCL2‐TLA	63126356	63126264	2269	105863148	105863114	JH6
TLA25	BM	No marker	BCL2‐TLA	63124949	63124866	3676	105914386	105914359	DH3‐3
TLA26	BM	No marker	BCL2‐TLA	63107660	63107635	20956	105863252	105863144	JH6
TLA27	BM	No marker	BCL2‐TLA	63125450	63125411	3175	105863239	105863130	JH6
TLA29	BM	No marker	BCL1‐TLA	63120103	63120041	8522	105886030	105885959	DH3‐3

In the FL group, BAM files alignment was possible only in six out of eight patients, while in two cases (TLA 40 and TLA 41), translocation peaks were observed but the breakpoint sequences were not identified due to the low coverage.

Otherwise, in the other 6 FL patients, analysis showed clonal reads aligned on chromosome 18 (genomic nucleotide positions spanned between 63097360 and 63126356) at 3′ end of MBR BCL2 locus (annotation: 63126264) (Figure 2b; Table 2). Interestingly, among the new TLA positive FL cases, two cases (TLA 25 and TLA 29) mapped in the region between MBR and MCR on the chromosome 18, (not investigated using classic BCL2/IGH PCR) and mated to the IGHD3‐3 region, defining unusual *t*(14; 18) translocations, not yet described in FL (Figure 3b).

### 3.4 TLA sequences validation as molecular markers

The new BCL1‐TLA and BCL2‐TLA markers were validated by ASO‐qPCR strategy.^25^

With ASO primer design, a sequence validation was possible in 14 out of 16 (86%) MCL patients. In two cases no validation was possible due to an uncommon sequence structure, leading to no target and polyclonal background amplification, respectively (data not shown).

In FL cases, five out of six (83%) BCL2‐TLA sequences were validated using the same ASO strategy. The BCL2‐TLA sequence validation failed in one sample (TLA 23), probably due to the close proximity but incomplete involvement of IGHJ6 region that prevented the optimal annealing of the IGHJ consensus primer (reads mapped 48bp at 3'side of IGHJ6; data not shown).

### 3.5 Tumor burden quantification and MRD monitoring by TLA molecular markers

Out of the 12 patients of the “double positive marker” MCL group, 10 could be evaluated for comparative MRD analysis by ASO q‐PCR, using both BCL1‐TLA and IGH as molecular markers. As mentioned before, two patients (TLA 9 and TLA 11) were excluded because the BCL1‐TLA sequences were not validated.

**FIGURE 4:**
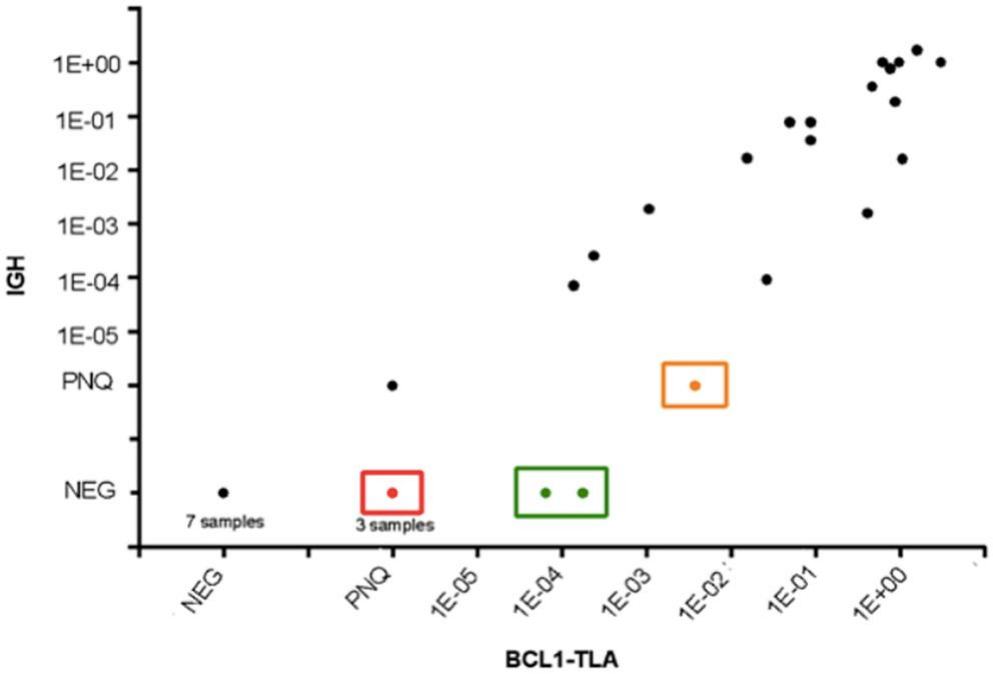
Correlation between *BCL1‐TLA* and *IGH* quantification. Major discordances were described as positive or PNQ for *BCL1‐TLA* and negative for *IGH* (red dots), positive BCL1‐TLA versus negative IGH sample (green dots). Minor discordance resulted as positive for *BCL1‐TLA* and PNQ for *IGH* (orange dot)

Overall, ASO q‐PCR data from 35 samples (10 diagnostic and 25 FU) showed good correlation between the new TLA molecular markers and the well standardized IGH rearrangements (Pearson's *R* = 0.7591, *p* < 0.0001, Figure 4). Notably, 28 samples showed concordant results (80%) (Figure 4, black dots). Discordances were described in six samples (17%): five major discordances were represented by three PNQ BCL1‐TLA versus negative IGH samples (Figure 4, red dots, 8%), two positive BCL1‐TLA versus negative IGH sample (Figure 4, green dots, 6%). Finally, one sample showed minor quantitative discordances, that is, positive for BCL1‐TLA and PNQ for IGH (Figure 4; orange dot; 3%).

**FIGURE 5:**
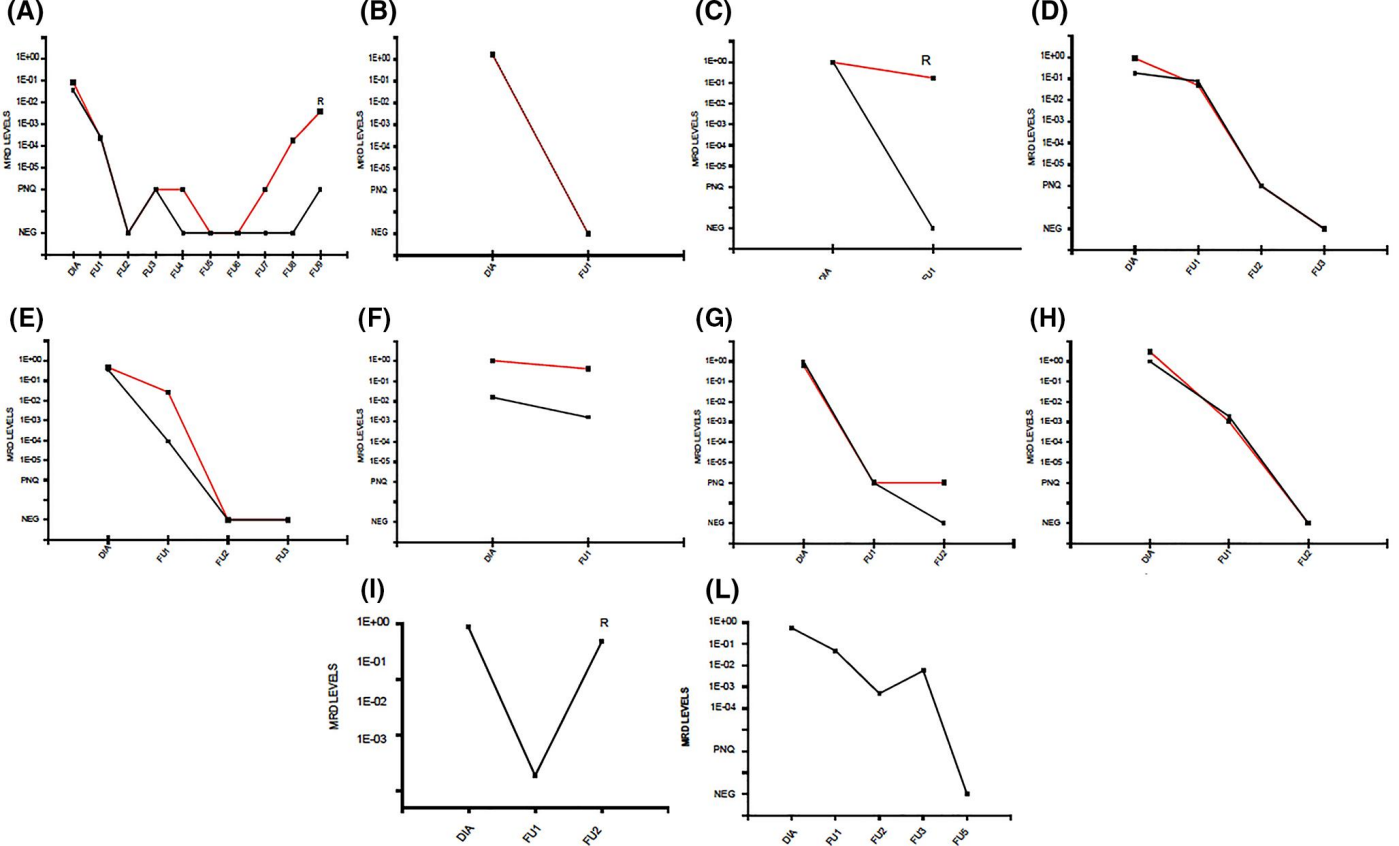
Minimal residual disease (MRD) trends by *BCL1‐TLA* (red line) and *IGH* (black line) in “double markers” mantle cell lymphoma patients with available FU samples postdiagnosis (panels A–I); MRD trends by *BCL2‐TLA* (black line) in follicular lymphoma patients (panel L–M)

Detailed comparison of MRD quantification by both BCL1‐TLA and IGH markers was described in eight patients with available follow‐up samples (Figure 5). Three patients (TLA 5, 7, and 19) showed totally superimposable MRD trends, with no relevant discordances between the two markers (panels B, D, and H). Differently, BCL1‐TLA determined higher MRD levels over IGH results in TLA 2‐FU2, TLA 3‐FU3/4/7/8/9, and TLA 12‐FU1 (panels C, A, and F). Moreover, minor differences in MRD quantification were described in TLA 8‐FU1 and TLA 10 (panels E and G).

Interestingly, in two patients (TLA 2 and TLA 3) BCL1‐TLA marker seemed to better predict clinical outcome than the standard IGH, detecting the MRD positivity in advance of at least one follow‐up (i.e. 6 months) prior to clinical relapse (Figure 5, panels C and A, respectively).

**FIGURE 6:**
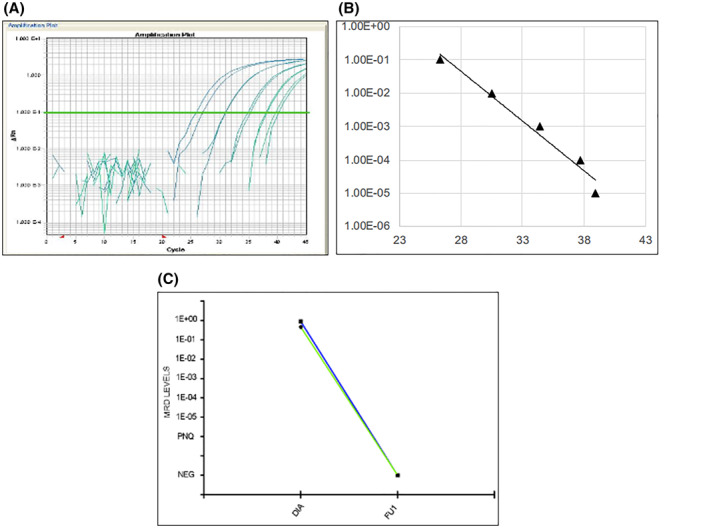
Targeted locust amplification 13 standard curve performances (panel A and B) and minimal residual disease trends in bone marrow (blue line) and peripheral blood (green line) follow ups targeting *BCL1‐TLA* (panel C)

Regarding the MCL “no marker” patients, TLA provided a new marker in four out of five cases, as previously reported. In all cases (TLA 13, TLA 16, TLA 20, and TLA 21) it was possible to validate the BCL1‐TLA sequence and to set a reliable standard curve (with an optimal sensitivity > 1E‐04). However, FU samples were available only for TLA 13, since only samples from marker positive patients should be collected, as per protocol. Actually, in this case the BCL1‐TLA was able to describe a deep MRD shrinkage after induction therapy, both in BM and PB FU samples (Figure 6).

Finally, in FL cases with available FU samples, MRD was monitored using BCL2‐TLA. In both TLA 26 and TLA 27, the experimental setting reached high level of sensitivity (1E‐04 and 1E‐05, Figure 5, panels I and L, respectively) and was nicely able to describe the MRD persistence in a primary refractory patient (i.e., TLA 26, see Figure 5, panel I).

## 4 DISCUSSION

This study describes the first application of the TLA technique in MCL and FL primary samples as a NGS tool to detect novel molecular markers for MRD analysis.

Our results can be summarized as follows:

1) TLA is a feasible marker screening approach in most MCL patients, both in BM and PB samples, able to successfully identify new BCL1/IGH breakpoint regions (BCL1‐TLA) in 16 out of 20 cases of our series (80%). TLA was promising also in FL series, in which a molecular marker (BCL2‐TLA) was detected in 8 out of 20 (40%) samples, previously defined as “no marker FL” by classic PCR;
2) In both series, some technical failures were registered probably due to low tumor infiltration of the tested samples (<5% by MFC).
3) The new detected *t*(11; 14) and *t*(18; 14) breakpoints mapped in regions different from the classical MTC, MBR, and MCR loci.
4) Most of the new identified molecular markers (14/16 of MCL and 4/5 of FL) were suitable for MRD analysis; failures in sequence validation were due to overlapping nucleotides, absence of patient‐specific *N* insertions, and incomplete involvement of the IGHJ6 region, not allowing an optimal ASO primer and probe setting.
5) The comparison between BCL1‐TLA and the well‐standardized IGH markers by ASO‐qPCR showed an overall good correlation in eight MCL patients.
6) In some cases, breakpoint‐based MRD tests showed superior performance over IGH‐based breakpoints.
7) Novel BCL1 and BCL2/IGH breakpoints were found in “no marker” MCL and FL cases, opening new possibilities for MRD assessment in these patients.

This study offers the proof of principle that TLA can identify novel fusion targets and structural variants in MCL and FL. Actually, TLA identified new BCL1/IGH breakpoints in the majority of patients of our series (80%), accounting for an overall increase in MRD marker detection rate up to 95% (19 out of 20 patients), by using both the standard IGH marker and the new BCL1‐TLA. Even more interesting are the preliminary results obtained from the FL series; the identification of a BCL2‐TLA in 8 out of 20 (40%) of FL patients without a molecular marker according to the classic PCR method suggested a potential increase of the number of patients to be included in future MRD analyses.

Moreover, TLA did not detect any translocation in healthy donors samples, thus providing information about specificity of the test.

Nevertheless, this NGS technology was not able to detect a molecular marker in two out of three FL patients with very low tumor burden (1E−05, that means 1 clonal cell out of 500,000 analyzed cells). These results underlined the TLA ability to detect molecular targets in diagnostic samples characterized by high tumor burden, including in a quantitative range of at least 1E−04.

Although the coupling of TLA marker screening with the standardized ASO‐qPCR approach allowed to generate sensitive and reliable MRD assays, in two cases the new breakpoint sequences were not suitable for MRD analysis. Nevertheless, BCL1‐TLA marker validation by ASO‐qPCR was performed successfully on diagnostic samples of 14 patients. Moreover, patients carrying a previously detected IGH rearrangement offered the possibility of a comparison between the standard molecular marker and the new BCL1‐TLA for MRD monitoring. Overall, a good concordance was achieved (R Pearson = 0.7591), confirming that the new marker is as reliable as the IGH for MRD purposes. Despite an overall good concordance, a few minor and major discordances were observed at different time points. Notably, in two cases (TLA 2 and 3), BCL1‐TLA seemed to predict clinical outcome even better than IGH, highlighting, in relapsing patients, a first MRD positive signal 6 and 12 months before the IGH reappearance, respectively. Consistently, the MRD monitoring by BCL2‐TLA in a FL patient was able to describe the persistently high tumor burden in a primary refractory patient, tracing a chemo‐resistant clone.

Some limitations of this study need to be addressed. First, the small number of analyzed cases: 20 MCL and 20 FL analyzed patients are clearly not enough to directly support large‐scale application of the TLA technique, and therefore, larger patients and samples cohorts are needed to validate these data. Second, the lack of FU samples in PCR no marker MCL and FL patients did not allow to properly evaluate the impact of BCL1‐TLA and BCL2‐TLA on MRD monitoring of patients carrying no standard marker. Nonetheless, based on present data, TLA is emerging as a feasible tool for marker screening in lymphoma, that might allow more patients to be monitored for MRD by classical ASO‐qPCR or digital droplet PCR assays,^26^ highly sensitive, and standardized approaches for MRD analysis. Moreover, TLA needs to be integrated with other NGS tools, as IGH amplicon based NGS method,^27^ to cover all the targetable markers for MRD detection.

In conclusion, this study is the proof of principle of the feasibility of TLA technology for MRD marker screening in MCL and FL patients. TLA significantly increased the marker identification rate in both disorders, especially in FL patients in which standard approaches allow MRD analysis only in up to 60% of cases. Moreover, the new “minor” BCL1/IGH breakpoints performed well as MRD markers in comparison to the standard IGH approach and allowed to assess MRD in patients lacking a classical molecular marker. Although some methodological issues still need to be addressed, TLA technology shows promising results, and its applications deserve to be more widely investigated in other hematological malignancies, too.

## CONFLICT OF INTERESTS

Petra Klous, Mehmet Yilmaz, Max van Min, and Erik Splinter are Cergentis employees. The other authors declare no conflicts of interest.

## AUTHOR CONTRIBUTIONS

All authors contributed to designing the study, performing the research, analyzing the data, and writing the paper.

### TRANSPARENT PEER REVIEW

The peer review history for this article is available at https://publons.com/publon/10.1002/hon.2864.

## Data Availability

The data that support the findings of this study are available from the corresponding author upon request.
